# Kyle Harper, *Plagues upon the Earth: Disease and the Course of Human History*. John Mokyr, series ed. *The Princeton Economic History of the Western World*

**DOI:** 10.1093/emph/eoac014

**Published:** 2022-04-18

**Authors:** Margaret Humphreys

**Affiliations:** Duke University, Durham, NC, USA

‘The history of disease is the history of migration and power, of poverty and prosperity, of progress and its unintended consequences. In short, our history as a species is inseparable from our strange and intimate connection with the parasites that have stalked our journey every step of the way’. Kyle Harper’s new history of infectious diseases views us as one more animal, never distinct from nature, subject to the rules of evolution and ecology. He nicely lays out the parasite’s approach of the human host: get in, multiply, get out. The story begins with *Homo erectus*, and ends in the modern world infested with coronavirus disease 2019 (COVID-19). In spite of such a portentous topic, his writing remains light and engaging.

Harper begins with the relentless evolution of microbes, especially viruses, the most successful and abundant entities in the biosphere. Human history arrives only as a miniscule recent event in a hugely long story. It was *H.erectus* who first tamed fire, and migrated out of Africa to occupy Asia and Europe. The species abundance of the equatorial region meant many foods, yes, but also many insects to carry disease. Our first disease risks came not from domestication, but from these mobile carriers, especially *Anopheles* and *Aedes* mosquitoes, as well as the Tse tse fly. Helminths rode comfortably in human guts. Humans who migrated to ‘Where the bloodsuckers aren’t’ (to quote the title of chapter 3) were more successful in population growth. By the end of the Pleistocene, there was something like 10 million *Homo sapiens* on earth. Our big brains, conquest of fire and travel had led to success.

It is only in the last 5% of our history as a species that humans left the hunter-gatherer life for settlement and agriculture. The Neolithic revolution (named after the first tools, crafted of stone) happened, Harper tells us, at least a dozen times in different places. Harper directly addresses the contentious historiography of the agricultural revolution, but then moves on to analyze how sedentary life changed the human disease environment. Here, human waste disposal became the biggest challenge to health. Fecal-oral gastrointestinal diseases, carried by dirty hands, contaminated water and the house fly, which proved so handy in carrying microbes on its feet.

The sedentary domus of the neolithic also encouraged diseases spread by respiratory droplets, noted by Harper in a chapter called ‘The sneezing ape’. As populations grew and became interconnected, diseases like measles, which immunize their hosts, could circulate in populations large enough (250 K) to allow their persistence.

From then on, every step humans took toward ‘civilization’ brought new disease threats with it. Domesticated animals did not so much bring diseases of their own, Harper argues, but rather served as intermediary reservoirs for wild animal diseases, often from rodents. Domesticated horses and other beasts of burden, pulling wheeled carts, increased trade and connections further, allowing microbes to travel across continents.

Such ‘progress’ in building towns and trade increased social stratification, which left signs of poverty and chronic disease imprinted in their populations’ bones. The Antonine plague (161+ AD, likely smallpox) and the Justinian Plague (561+ AD, an extinct form of bubonic plague) were both major mortality events—the first pandemics of the Western World—that ultimately contributed to the fall of the Roman Empire.

Bubonic plague recurred in earnest in 1348, snuffing out half(?) the population of Europe in just a few years. In its wake came the first European contact with the Americas, resulting in massive population declines as European microbes coupled with harsh treatment caused a precipitous decline of local American populations. The population crash of Mexico in the 16th century was one of the most extreme demographic events attested to by history, when 60–80% of the population perished. One mass grave from that period and place has yielded the DNA of *Salmonella enterica paratyphi C*, a gut organism that causes paratyphoid fever. Disseminated through fecal-oral spread, this may have been a fairly innocuous traveler for the Europeans who brought it, but the cause of a malignant pandemic to the Native Americans (271).

From here, Harper expands on the Caribbean disease environments created by the enslavement of Africans (along with their microbes and mosquitoes), the incarceration of such slaves in the new world to raise sugar and cotton, and the subsequent inauguration of a new disease environment featuring vector-borne diseases. Yellow fever, malaria and hookworm were thus able to move from Africa to the Americas. Harper emphasizes that ‘infectious diseases were an integral factor in the patterns of commerce, colonization, settlement, and slavery around the Atlantic’ (288). He recognizes the ‘interrelationships between agrarian systems, demographic dynamics, social structures, and microbial ecologies’ (289). In the cooler northern colonies where settler colonialism prevailed, population numbers exploded, a phenomenon noted by Thomas Malthus as the archetypal example of maximal human reproductive rates. The Caribbean, and the subtropical American south, on the other hand, became, to varying degrees, environments of disease and early mortality.

After reviewing the causes of high mortality in Europe during the 17th and 18th centuries (smallpox, typhus, plague), Harper dives into the ‘great escape’, beginning with the industrial revolution and ending with a doubling of life expectancy by the 20th century (at least in the west). He notes the population cost of industrialization, including the arrival of cholera from India and the fecal-oral fevers that plagued the crowded slums housing the workers for those factories. He also pauses to discuss the emergence of agricultural diseases such as hog cholera, rinderpest, Texas cattle fever and various animal influenzas.

The book closes with the key triumph of the 20th century: the conquest of infectious diseases made possible by insecticides, vaccines, plumbing and antibiotics. This is, he says, ‘is one of the unambiguously great accomplishments of our species’ (469). Certainly, it is one of the most important factors in the rise of human populations to number in the billions. While his triumphalism was a bit subdued by the arrival of COVID-19 as the book was in progress, still the rapid production of a vaccine supports his overall narrative. Yes, new plagues will continue to arise, especially as humans increase contact with wild rodent populations, but humans are also shielded from total annihilation by our scientific tools.

This is a wonderful book for class use. Although not yet available in paperback, Princeton University Press has priced the hardback reasonably, and there are Kindle and audiobook versions already. The scientific ideas are presented with clarity and should be accessible to most college students. Historians of disease who approach the topic with a scientific perspective are unlikely to find something novel here, however. The book’s strength is its encyclopedic approach, with 150+ pages of references, bibliography and a handy checklist of major identified species of human pathogens.

Harper’s *Plagues upon the Earth* expands upon a long traditions of works on the history of epidemic disease. Most relevant are C.-E. A Winslow, *The Conquest of Epidemic Disease* (1943); Alfred Crosby, *The Columbian Exchange* (1973); and William H. McNeill, *Plagues and Peoples* (1977), in addition to a rich literature that focuses on particular times and places. (Disease history is a sexy topic, especially when the gruesome is emphasized on the jacket.) Aside from his impressive synthesis of human experience across the millennia, Harper’s wielding of concepts and discoveries from ecology, evolution and DNA evidence updates these older accounts for the modern era. More than a hundred pages of notes and references direct the reader to further, specific topics.

It can be argued that he skimps a bit on the last two centuries. Harper is, after all, a historian of ancient Rome. And, probably inevitably, the book focuses on Europe and the ‘unification of the tropics’ with only a few pages on the disease history within Asia and India. He recognizes the importance of Asian diseases coming to the west, such as the movement of plague via the Silk Road, or Indian cholera by merchant vessels. But there is much more to be done about the evolution of disease climates within these vast areas.

Still, no other volume has so clearly chronicled the significance of infectious disease ecology in the human experience, from *H.erectus* to 21st-century humans. Far from Winslow’s confidence in ‘The Conquest of Epidemic Disease’, we are strongly reminded here that the encounter continues, and we remain vulnerable to the next ingenious microbe that threatens our confident society.

